# Bed bugs and possible transmission of human pathogens: a systematic review

**DOI:** 10.1007/s00403-016-1661-8

**Published:** 2016-06-13

**Authors:** Olivia Lai, Derek Ho, Sharon Glick, Jared Jagdeo

**Affiliations:** 1 Keck School of Medicine, University of Southern California, Los Angeles, CA USA; 2 Dermatology Service, Sacramento VA Medical Center, Mather, CA USA; 3 Department of Dermatology, State University of New York Downstate Medical Center, Brooklyn, NY USA; 4 Department of Dermatology, University of California Davis, Sacramento, CA USA

**Keywords:** Bed bugs, Public health, Pathogens, Infectious disease

## Abstract

The global population of bed bugs (*Cimex lectularius* and *Cimex hemipterus*, family Cimicidae) has undergone a significant resurgence since the late 1990s. This is likely due to an increase in global travel, trade, and the number of insecticide-resistant bed bugs. The global bed bug population is estimated to be increasing by 100–500 % annually. The worldwide spread of bed bugs is concerning, because they are a significant socioeconomic burden and a major concern to public health. According to the United States Environmental Protection Agency, bed bugs are “a pest of significant health importance.” Additionally, 68 % of U.S. pest professionals reported that bed bugs are the most challenging pest to treat. Upwards of 45 disease pathogens have been reported in bed bugs. Recent studies report that bed bugs may be competent vectors for pathogens, such as *Bartonella quintana* and *Trypanosoma cruzi*. However, public health reports have thus far failed to produce evidence that major infectious disease outbreaks have been associated with bed bugs. Since many disease pathogens have previously been reported in bed bugs and the worldwide bed bug population is now drastically increasing, it stands to reason to wonder if bed bugs might transmit human pathogens. This review includes a literature search on recently published clinical and laboratory studies (1990–2016) investigating bed bugs as potential vectors of infectious disease, and reports the significant findings and limitations of the reviewed studies. To date, no published study has demonstrated a causal relationship between bed bugs and infectious disease transmission in humans. Also, we present and propose to expand on previous hypotheses as to why bed bugs do not transmit human pathogens. Bed bugs may contain “neutralizing factors” that attenuate pathogen virulence and, thereby, decrease the ability of bed bugs to transmit infectious disease.

## Introduction

The global population of bed bugs (*Cimex lectularius* and *Cimex hemipterus*, family Cimicidae) has undergone a significant resurgence since the late 1990s [1, 2, 13, 14, 17, 26, 36, 37, 47]. This is likely due to an increase in global travel, trade, and the number of insecticide-resistant bed bugs [11, 21]. In 2014, the number of international travelers reached 1.1 billion (leisure tourists accounted for 53 %) and is estimated to reach 1.8 billion by 2030 [45]. Travelers are at a particular risk for infestation, as bed bugs have been detected in aircraft, boats, trains, and hotels [12]. The global bed bug population is estimated to be increasing by 100–500 % annually [3]. In one nationwide survey, 99.6 % of United States (U.S.) pest professionals reported that they have treated bed bugs in the past year, and 68 % of U.S. pest professionals reported that bed bugs are the most challenging pest to treat [30].

Bed bugs are a significant socioeconomic burden. Complete eradication of bed bugs is challenging as bed bugs are very mobile and can travel extensively to neighboring units [9]. The estimated cost of disinfecting a house with standard insecticide and replacing infested belongings, such as clothes and bedding, is approximately $2500–$3000 (USD) per infestation [11]. For commercial and industrial workplaces, infestations may cost upwards of millions of dollars [35], and the healthcare industry is no exception. A study reported that 58 % of U.S. pest professionals encountered infestations in nursing homes, 36 % in hospitals, and 33 % in physician offices [30]. Additionally, entire hospital wards have been shut down due to infestations [4, 41].

Published evidence supports that bed bugs are experiencing a global resurgence and that bed bugs once thought to be native to certain geographic locations have been found in other parts of the world [11, 42]. For example, in 2014, *C. lectularius* was detected for the first time in the Chilean province of Magallanes, which is the southernmost record for this species in South America [16]. *Cimex hemipterus,* the bed bug indigenous to the tropics and subtropics, has been found in the United Kingdom, and is representative of the global spread of bed bugs [11]. *Cimex lectularius* and *C. hemipterus* are two species that primarily feed on humans (in addition to domestic animals) [13, 18], and a global spread of these two bed bug species may translate to widespread infestations and may also act as a route of human pathogen transmission. Recently, the media and the medical community have been concerned about this very possibility, and the amount of media attention focused on bed bugs has increased [11, 13, 39].

Bed bugs are a major concern to public health. According to the U.S. Environmental Protection Agency, bed bugs are “a pest of significant health importance,” and bed bugs have been reported to carry more than 40 microorganisms in the stomach, feces, exoskeletons, and/or saliva [13, 46]. Recent studies reported that bed bugs may act as competent vectors for pathogens, such as *Bartonella quintana* and *Trypanosoma cruzi*, the causes of trench fever and Chagas disease, respectively [23, 24, 37]. However, public health reports have thus far failed to produce evidence that major infectious disease outbreaks have been associated with bed bugs. Additionally, other members of the family Cimicidae are competent vectors for arboviruses for birds and, also, likely for wild bats [1, 14]. Since many disease pathogens have previously been reported in bed bugs and the worldwide bed bug population is now drastically increasing, it stands to reason to wonder if bed bugs might transmit human pathogens.

This review includes a literature search on recently published clinical and laboratory studies (1990–2016) investigating bed bugs as potential vectors of infectious disease, and reports the significant findings and limitations of the reviewed studies. Also, we present and propose to expand on previous hypotheses as to why bed bugs do not transmit human pathogens.

## Methods

### Systematic search strategy and data extraction

**Fig. 1:**
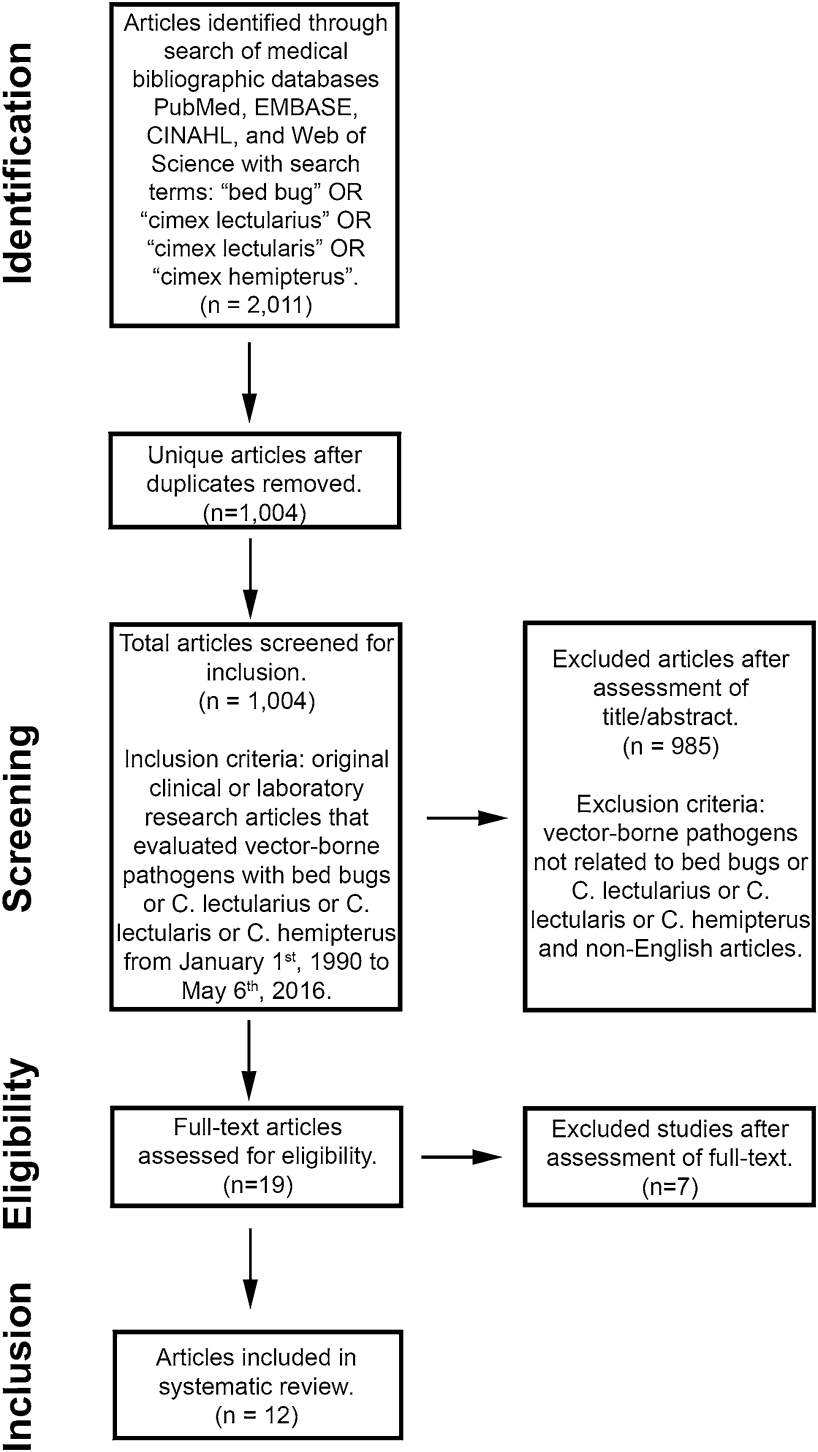
Schematic of literature search strategy based upon the preferred reporting items for systematic reviews and meta-analyses [PRISMA] guidelines [29]

We employed the following literature review search strategy: on May 6, 2016, we systematically searched the computerized medical bibliographic databases PubMed, EMBASE, CINAHL, and Web of Science with the following search terms: “bed bug” OR “cimex lectularius” OR “cimex lectularis” OR “cimex hemipterus” (see Fig. 1 for schematic of literature search strategy based upon the preferred reporting items for systematic reviews and meta-analyses [PRISMA] guidelines) [29]. The relevant articles that met the following criteria were selected for inclusion: original clinical or laboratory research articles that evaluated vector-borne pathogens with bed bugs or *C. lectularius* or *C. lectularis* or *C. hemipterus* from January 1, 1990 to May 6, 2016. Exclusion criteria included: vector-borne pathogens not related to bed bugs or *C. lectularius* or *C. lectularis* or *C. hemipterus* and non-English articles. Information on study type, bed bug genus/species, pathogen(s), and significant findings of published reports was extracted.

## Results

### Bed bugs as potential vectors of infectious diseases

A total of 2011 articles were returned from our search terms. After removal of duplicates, 1004 articles were screened for titles, abstracts, and/or full-texts, and 12 articles were found to be suitable for our review. These articles are summarized in Table 1 and discussed in the following section. To date, no published study has demonstrated a causal relationship between bed bugs and infectious disease transmission in humans [5, 6, 8, 19, 20, 23, 24, 26, 28, 32–34, 36, 37, 40]. Of note, animal studies reported that other members of the family Cimicidae [excluding *C. lectularius* (the common bed bug) and *C. hemipterus* (the tropical bed bug), which are the two Cimicidae members that are most associated with humans] are competent vectors for birds and likely wild bats, but we found no evidence that suggests that *C. lectularius* and *C. hemipterus* are competent vectors for the transmission of infectious diseases in animals [1, 14].

**Table 1:** Published reports of vector-borne pathogens with bed bugs (1990–2016)

Authors	Year	Study type	Genus/species	Pathogen(s)	Clinical/laboratory significant findings
Leulmi et al. [23, 24]	2015	Experimental	*C. lectularius*	*Bartonella quintana*	Demonstrated for the first time that bed bugs can acquire and maintain *B. quintana* organisms for more than 2 weeks and then release viable *B. quintana* organisms into their feces. Researchers observed the transmission of the bacterium to bed bug eggs as well as to L1 and L2 larvae. Since the bacterium was found to be localized in the digestive tract and not in the ovary, the authors of this paper suggested that the transmission of the bacterium to bed bug progeny may, in fact, be due to vertical non-transovarial and/or horizontal transmission
Saenz et al. [36]	2013	Report/experimental	*C. lectularius*	*Burkholderia multivorans*	Five bed bugs from four different apartments of an elderly housing building in North Carolina contained DNA sequences that corresponded to *B. multivorans*, an important pathogen in nosocomial infections that was not previously linked to an arthropod vector
Salazar et al. [37]	2015	Experimental	*C. lectularius*	*Trypanosoma cruzi*	Reported efficient and bidirectional transmission of *T. cruzi* between mice hosts and bed bugs in a laboratory environment through cohabitation and the application of feces to broken host skin
Goddard et al. [19]	2012	Experimental	Did not specify	*Rickettsia parkeri*	Two adult bed bugs were IFA and PCR positive for rickettsia-like organisms. These results indicate that remnants of *R. parkeri* survived in the bed bugs for 2 weeks, but the viability of the organisms in these two specimens could not be determined
Jupp et al. [20]	1991	Experimental	*C. lectularius*	HBV	HBV-infected bed bugs did not transmit HBV to chimpanzees
Mayans et al. [28]	1994	Intervention	*C. lectularius*	HBV	Insecticide spraying of the child’s dwelling was highly effective for reducing exposure to bed bugs, but there was no effect on HBV infection
Blow et al. [6]	2001	Experimental	*C. lectularius*	HBV	HBV was passed transstadially through one molt, was shed in fecal droplets for up to 35 days after the infectious blood meal, but was not passed transovarially. In bed bugs inoculated intrathoracically, HBV was detected for 21 days post-inoculation
Silverman et al. [40]	2001	Experimental	*C. lectularius*	HBV, HCV	Bed bugs and their excrement remained HBV DNA-positive throughout 54 days of testing. No HCV RNA was detected in bed bugs after feeding on an infectious meal
Lowe et al. [26]	2011	Report	*C. lectularius*	MRSA, VRE	Recovered MRSA and VRE from bed bugs in Vancouver, British Columbia
Barbarin et al. [5]	2014	Experimental	*C. lectularius*	MRSA	Results indicated that while the bed bug midgut is a hospitable environment for MRSA, the bacterium does not survive for longer than 9 days within the midgut, which suggests that bed bug transmission of MRSA is highly unlikely
Cockburn et al. [8]	2013	Experimental	*C. lectularius*	Non-pathogenic skin bacteria	Bacteria found commonly on human skin are closely associated with bed bugs and do not pose a risk to human health
Reinhardt et al. [34]	2005	Experimental	*C. lectularius*	*Penicillium chrysogenum, Stenotrophomonas maltophilia, Enterobacter hormaechei, Bacillus licheniformis, Staphylococcus saprophyticus*	No microbes were isolated from the piercing and sucking mouthparts. Consequently, the epidemiological significance of bed bugs carrying externally attached microbes is likely minimal

For a comprehensive list of all published original articles with bed bugs as potential vectors of infectious disease, please refer to these references [13, 48]

*HBV* hepatitis B virus, *HCV* hepatitis C virus, *IFA* immunofluorescence assays, *MRSA* methicillin-resistant *Staphylococcus aureus*, *PCR* polymerase chain reaction, *VRE* vancomycin-resistant *Enterococcus*

## Discussion

Based upon published evidence that we reviewed, there are no reports of bed bugs acting as infectious disease vectors in humans for *B. quintana, Burkholderia multivorans*, *T. cruzi, Rickettsia parkeri*, hepatitis B virus, hepatitis C virus, methicillin-resistant *Staphylococcus aureus*, vancomycin-resistant *Enterococcus*, *Penicillium chrysogenum*, *Stenotrophomonas maltophilia, Enterobacter hormaechei, Bacillus licheniformis,* and *Staphylococcus saprophyticus*. Although bed bugs may act as phoretic vectors (solely for transport) for these pathogens, there were no confirmed cases of human disease transmission (Table 1). Some of the aforementioned studies do suggest that pathogens, such as *B. quintana* and *T. cruzi*, may survive in bed bugs under laboratory conditions. It may be possible that because the insect vectors for *Bartonella* and *Trypanosoma* (the human body louse and triatomine bugs, respectively) are similar to bed bugs in that all of these are insects that feed on human blood, *B. quintana* and *T. cruzi* may be more prone to being transmitted by bed bugs due to vector similarity. Additional studies on other, similarly arthropod-borne pathogens can be helpful as they may help support or refute the hypothesis that bed bugs have the potential to transmit human pathogens.

Prior reports have also examined the ability of bed bugs to act as vectors for high profile infectious agents, such as human immunodeficiency virus, and for other pathogenic agents. For instance, in 2009, Goddard et al. reported that no known study has showed vector competence, or the ability to acquire, maintain, and transmit any infectious agents, for bed bugs [18]. Goddard et al. also reported that there is a lack of evidence for disease transmission by bed bugs [18]. Similarly, Delaunay et al.’s article in 2011 stated that there was no published evidence that bed bugs can transmit pathogens [13]. In 2012, Doggett et al. reported that no proven evidence exists to suggest that either bed bugs are competent vectors of any pathogen [14]. In 2015, Zorrilla-Vaca et al. reported that there is not sufficient evidence (since the 1940s) to confirm that bed bugs can transmit human pathogens [48]. Similarly, this review was unable to identify definitive evidence that bed bugs can transmit infectious diseases to humans.

Despite the fact that there is not yet definitive evidence that exists that bed bugs can act as vectors of human pathogens, studies, such as the ones that showed that pathogens, such as *B. quintana* and *T. cruzi*, may survive in bed bugs under laboratory conditions, are worrisome. There remains a possibility that bed bugs may possibly be able to transmit some human diseases.

**Fig. 2:**
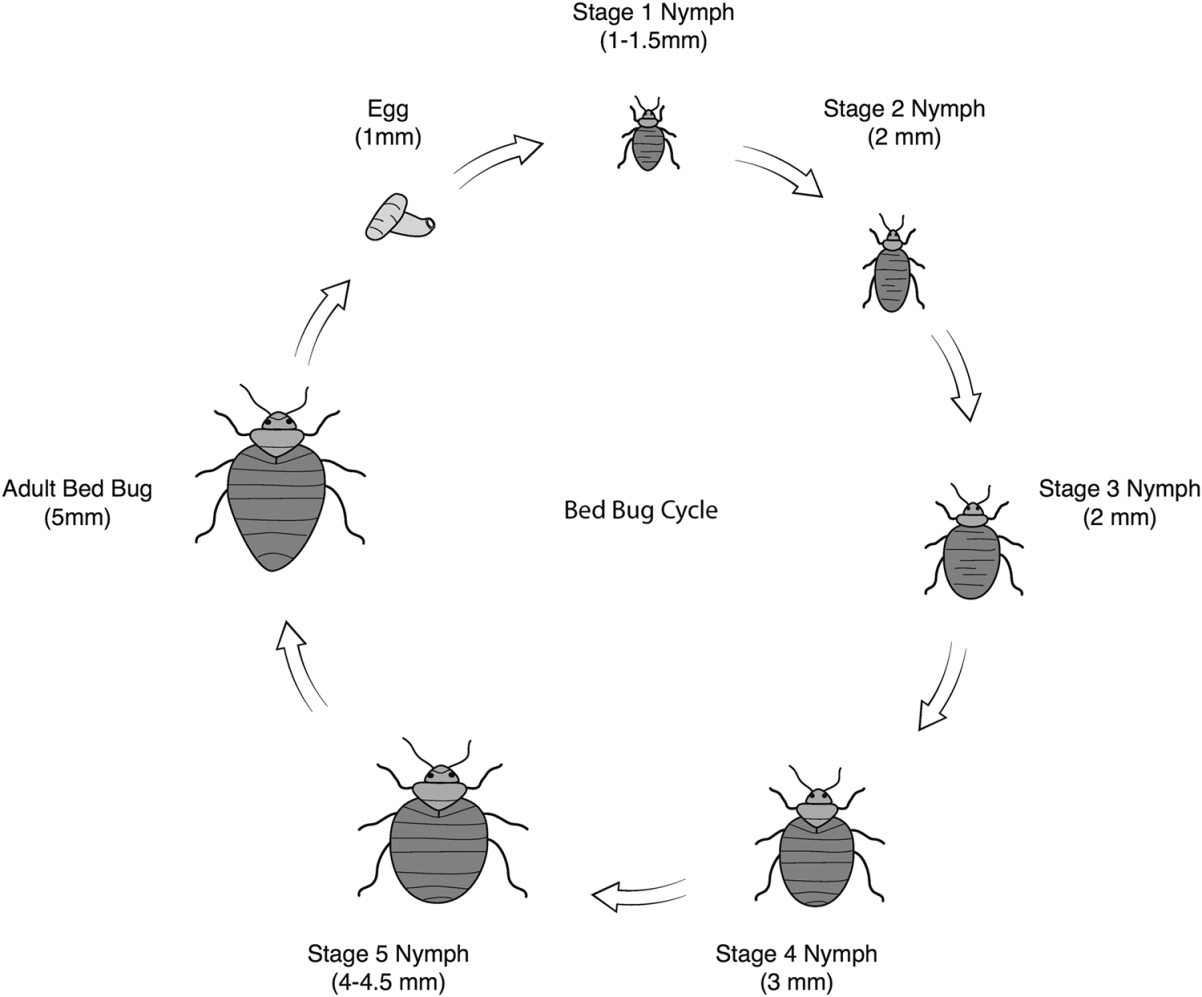
Female bed bugs lay several eggs. After 4–12 days, these eggs hatch into first instar nymphs. The bed bug goes through five nymphal stages prior to becoming an adult, and each molt requires a blood meal. After 6–8 weeks, the nymphs will become adults, and infestations may involve bed bugs in every stage of life. Adult bed bugs live for around 6–12 months and can survive for long periods of time without feeding [7] (Photo courtesy of Frank Fasano, SUNY Downstate Medical Center illustrator)

**Fig. 3:**
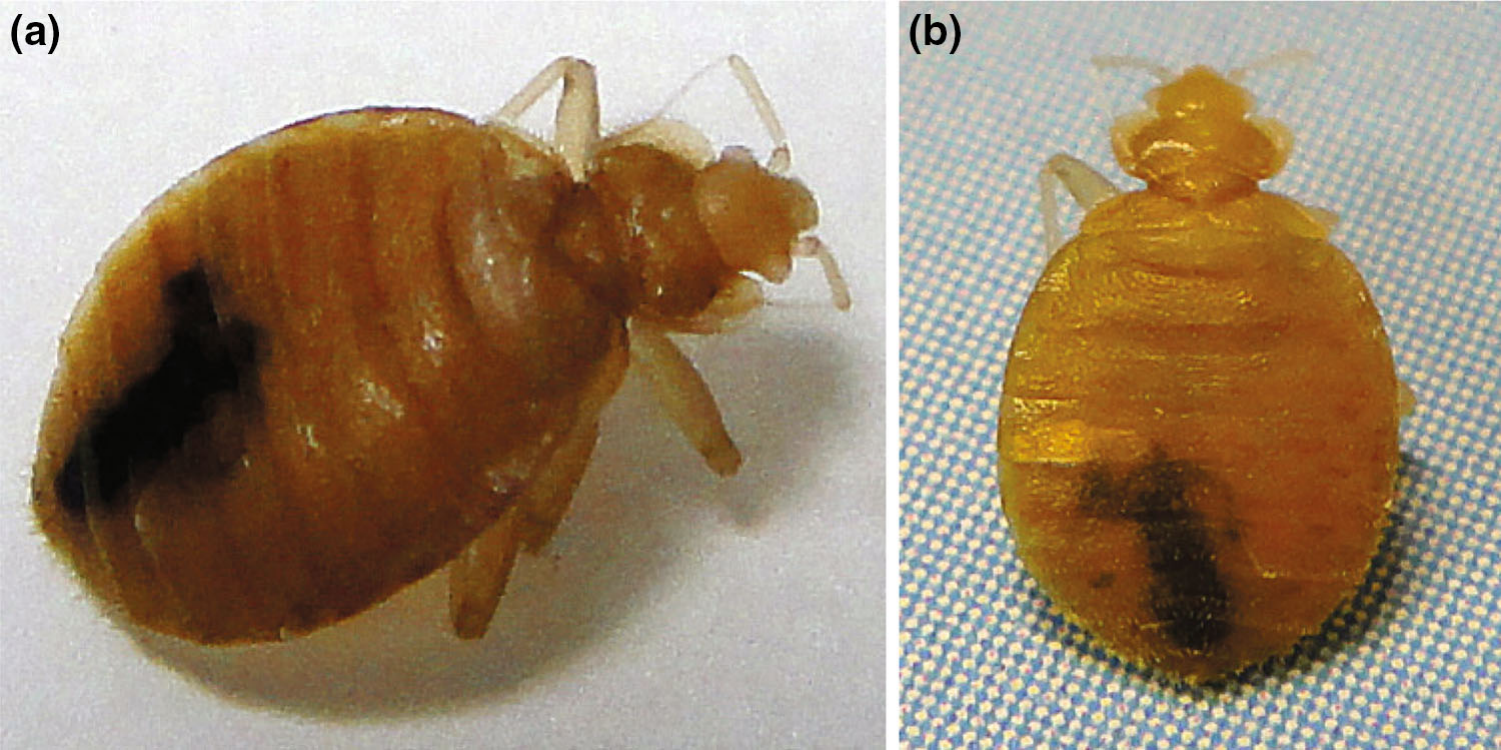
**a**, **b** The bed bug is a wingless, hematophagous insect with an oval shaped body and a pyramidal head. The adult bed bug is 5–7 mm in length when unfed, and can increase up to 50 % in size after feeding. Bed bugs belong to the family Cimicidae, which consists of (at least) 23 genera and 91 species. *C. lectularius* (the common bed bug) and *C. hemipterus* (the tropical bed bug) are most associated with humans

Additionally, since bed bugs are already known to be a socioeconomic burden and a major concern to public health, it is important that both physicians and the general public are aware of how bed bugs can appear at different stages of their life cycle (see Fig. 2 for details on the bed bug life cycle and Fig. 3a, b for morphological details about bed bugs). The importance of recognizing bed bugs cannot be understated, as one article reported that an elderly patient presented for medical treatment of “erythematous papules,” but did not alert the physician of “insects seen around the house” (later proven to be bed bugs) until four months later, because the patient did not realize the insects and the erythematous papules were related [22].

Early recognition and prevention of bed bug bites can translate to more efficient medical treatment and a higher likelihood of successful bed bug eradication. Bed bug bites can be distributed in a clustered arrangement or in a sequential, linear arrangement, and this arrangement is colloquially referred to as the “breakfast, lunch, and dinner” pattern [25]. The bed bug bites generally present as 2–5 mm pruritic, erythematous papules with a central punctum often on the limbs [10, 15, 18, 38]. Bed bug bites may be complicated by excoriations and secondary infections [18, 44], and rare systemic reactions, such as anaphylaxis, have also been reported [18, 43]. Lastly, if bed bug bites and infestations are accurately reported, the relationship between bed bugs and the transmission of infectious diseases in humans may be better understood.

Although many different pathogens have been detected in or on bed bugs, there is presently no published evidence that supports the ability of bed bugs to successfully transmit human pathogens. One hypothesis that may explain this seemingly contradictory finding is based on the fact that bed bugs are the only hematophagous arthropods that both feed on humans and mate by traumatic insemination. Traumatic insemination results in the repeated introduction of pathogens and repeated immune stimulation in the female bed bug. Traumatic insemination shortens the female bed bug lifespan and may lead to the increased natural selection of bed bugs who have more active immune systems. As a result, the survival and viability of pathogens maintained within bed bugs may be attenuated [13]. Additionally, bacteriolytic activity in bed bug ejaculate and hemolymph has also been reported [31]. It may be that immune-active substances in bed bug ejaculate and hemolymph may also contribute to the attenuated virulence of pathogens that are carried by bed bugs. A different hypothesis emerges from the discovery that bed bug saliva may contain proteases, lysozymes, and other potentially antimicrobial peptides, and that contact with bed bug saliva may, therefore, decrease the virulence of potential pathogens that are localized in bed bug salivary glands or that are transmitted through bed bug bites [14].

Since bed bug saliva, hemolymph, and ejaculate may contain compounds that attenuate pathogen virulence, these studies suggest that bed bugs may possibly possess intrinsic “neutralizing factors” in their bodies. These neutralizing factors may make it possible for bed bugs to carry, but not transmit, a variety of human pathogens. If these various “neutralizing factors” can be identified and isolated, it may improve the ability of scientists to better understand certain pathogens and prevent transmission of a variety of infectious diseases. This may be an important area for future research.

## Limitations

Inherent limitations exist with both clinical and basic science research studies on bed bugs. For instance, pathogens may behave very differently in laboratory conditions (ex vivo and in vitro) when compared to real-world conditions (in vivo). Some studies may also encounter difficulties in collecting a representative sample of bed bugs from a large, diverse population of bed bugs. Additionally, it may be too early to conclude that bed bugs cannot act as vectors of human infectious diseases. Although studies have reported that bed bugs are unable to transmit viral pathogens, such as hepatitis B virus and human immunodeficiency virus, arthropod-borne viral pathogens (such as Fort Morgan virus, Buggy Creek virus, and Kaeng Khoi virus) have not been specifically investigated. These arthropod-borne viral pathogens are known to have transmission cycles that involve insect vectors, and may be more likely than non-arthropod-borne viral pathogens to be transmitted by bed bugs [1].

Additionally, the clinical presentations of bed bug bites can vary, and occurrences of disease transmission in humans from bed bugs may, therefore, be underreported due to difficulties in identifying bed bugs as the causal agent. Not only are bed bug bites difficult to recognize, but disease surveillance in many developing countries is also often quite limited. These countries may lack strong public health systems [27]. Accurate information regarding bed bugs and the transmission of human pathogens, therefore, is likely to be lacking in developing countries.

Future high-quality bed bug research studies may improve our understanding of bed bugs as potential vectors of infectious disease in humans. Researchers may consider conducting experiments under a wide range of conditions, such as animal models and ex vivo human skin models, to substantiate and validate any positive findings. Additionally, experimental conditions for research experiments should closely replicate real-world settings. Future investigations of the relationship between bed bugs and additional pathogens, especially viral pathogens known to have transmission cycles that involve insect vectors, as well as increased recognition of bed bug bites and increased surveillance of bed bugs in developing countries, may potentially reveal undiscovered insights into bed bug-mediated human disease transmission.

## Conclusion and future directions

Bed bugs are a socioeconomic burden and a significant concern to public health. This review reports on the recent literature, summarizes the significance of bed bugs as potential vectors of infectious disease, and expands on previous hypotheses as to why bed bugs do not transmit human pathogens. Bed bugs may contain “neutralizing factors” that attenuate pathogen virulence and, thereby, decrease the ability of bed bugs to transmit infectious disease. To date, no published clinical or epidemiologic data have demonstrated a causal relationship between bed bugs and infectious diseases in humans. Due to a paucity of available studies in the published literature, additional studies may help to elucidate whether bed bugs can indeed transmit human pathogens.
